# Remember Hard But Think Softly: Metaphorical Effects of Hardness/Softness on Cognitive Functions

**DOI:** 10.3389/fpsyg.2016.01343

**Published:** 2016-09-12

**Authors:** Jiushu Xie, Zhi Lu, Ruiming Wang, Zhenguang G. Cai

**Affiliations:** ^1^Center for Studies of Psychological Application, School of Psychology, South China Normal UniversityGuangzhou, China; ^2^Guangdong Provincial Key Laboratory of Mental Health and Cognitive Science, South China Normal UniversityGuangzhou, China; ^3^School of Psychology, University of East AngliaNorwich, UK; ^4^Department of Experimental Psychology, University College LondonLondon, UK

**Keywords:** embodied cognition, metaphor, tactile sensation, hardness, softness, memory, creativity, cognitive function

## Abstract

Previous studies have found that bodily stimulation, such as hardness biases social judgment and evaluation via metaphorical association; however, it remains unclear whether bodily stimulation also affects cognitive functions, such as memory and creativity. The current study used metaphorical associations between “hard” and “rigid” and between “soft” and “flexible” in Chinese, to investigate whether the experience of hardness affects cognitive functions whose performance depends prospectively on rigidity (memory) and flexibility (creativity). In Experiment 1, we found that Chinese-speaking participants performed better at recalling previously memorized words while sitting on a hard-surface stool (the hard condition) than a cushioned one (the soft condition). In Experiment 2, participants sitting on a cushioned stool outperformed those sitting on a hard-surface stool on a Chinese riddle task, which required creative/flexible thinking, but not on an analogical reasoning task, which required both rigid and flexible thinking. The results suggest the hardness experience affects cognitive functions that are metaphorically associated with rigidity or flexibility. They support the embodiment proposition that cognitive functions and representations can be grounded in bodily states via metaphorical associations.

## Introduction

Traditional theories of cognition take a dualist approach to the mind and the body. To use a computer metaphor, the mind and its cognitive functions are equated with the operational system and the algorithms whereas the body is assumed to function like the hardware (e.g., the keyboard and monitor) (Pylyshyn, 1984; Neisser, 2014). This approach implies that the mind is independent of the body, just as a software package is independent of a computer’s physical settings. Such a dualist assumption has been challenged by a more recent embodied cognition approach, which assumes that the body plays a key role in shaping how the mind works (Lakoff and Johnson, 1980b; Kövecses, 2003; Barsalou, 2008; Landau et al., 2010; Meier et al., 2012). Over the past decade, research has converged to suggest that knowledge represented in our long-term memory consists of bodily or sensorimotor experiences acquired from interactions with the physical world (Mandler, 1992; Barsalou, 2003). From the beginning of human life, representations and understanding of the outside world are developed through interactions between sensorimotor systems and the environment (Smith, 2005). Thus, higher cognitive functions, such as memory, language, and reasoning, are grounded in perceptual, motor, and introspective contents instead of symbolic representations (Lakoff and Johnson, 1980b; Gallese, 2005, 2007; Barsalou, 2008; Barsalou, 2009, 2010; Gallese and Sinigaglia, 2011).

Cognitive functions can be embodied in two ways: sensorimotor embodiment and metaphorical association. First, when an event is experienced, the underlying sensorimotor states are partially stored. The representations underlying cognitive functions are simulations of past modal experiences rather than amodal symbols. When knowledge of the event is activated later, these sensorimotor states are partially stimulated. Thus, the compatibility of bodily states and cognitive states influences individuals’ performance effectiveness (Barsalou, 1999; Barsalou et al., 2003). This perspective of simulations has been verified in a variety of studies demonstrating an interaction between sensorimotor information and cognitive processing. For instance, conceptual processing evokes multimodal perceptual information associated with a concept’s referent (Solomon and Barsalou, 2001; Pecher et al., 2003, 2004; Solomon and Barsalou, 2004), and language comprehension makes use of sensorimotor simulation of the event that is being described in a sentence (Stanfield and Zwaan, 2001; Zwaan et al., 2002; Glenberg and Kaschak, 2003). A second way to achieve bodily grounding for cognitive functions is via metaphorical association, a mechanism that has been hypothesized to provide a “scaffold” for the acquisition and formation of new knowledge, especially that of abstract domains, such as time (Mandler, 1992; Lakoff and Johnson, 1999; Barsalou, 2003; Williams et al., 2009). For instance, time metaphorically recruits spatial representational vocabulary for its processing and representation (e.g., a time point can be *before* or *after* another, and duration can be *long* or *short*). Furthermore, the perception of time has been shown to be influenced by concurrent spatial information (Boroditsky, 2001; Casasanto and Boroditsky, 2008; Cai et al., 2013; cf. Cai and Connell, 2015).

One source of sensorimotor information that has been reported to provide bodily grounding for cognitive functions via metaphorical association is the sense of touch (Gallace and Spence, 2010). Among all the sensory modalities, the tactile sense is the first sense to develop and the last to fade (Gallace and Spence, 2010). Hence, during one’s lifetime, tactile sensation accumulates enormous amounts of information to support cognitive functions. Specifically, many studies have revealed that tactile sensation can modulate higher-level cognitive functions (Brunye et al., 2012; Maister et al., 2013). The tactile sensation of heaviness, for instance, can provide grounding for abstract domains, such as value, importance, and confidence by way of a metaphorical association with “weight” (e.g., more important/valuable things carry more weight). In previous studies, people tended to judge a foreign currency as more valuable (Jostmann et al., 2009), to express more self-confidence (Jostmann et al., 2009), and to rate job candidates as more suitable (Ackerman et al., 2010) when they were carrying a heavier physical load (e.g., holding a heavier clipboard). These findings suggest that tactile sensation might modulate participants’ social- and self-perception. The tactile sensation of heaviness has also been found to affect participants’ cognitive function. Kaspar and Vennekotter (2015) found that participants performed worse in a riddle task (e.g., re-arranging “cctiat” into “tactic”) if they concurrently held a heavier clipboard, presumably because a heavy load induced a “task-is-difficult” mindset, thereby hindering participants’ performance. Indeed, tasks appear to be more difficult for people who are carrying a heavy load; e.g., people carrying a heavier backpack tend to judge a hill’s slope to be steeper (Proffitt, 2006).

Further evidence concerning the effect of tactile sensations on social perception and cognitive function has been found in studies using the tactile sensation of hardness. Hardness is (in English at least) metaphorically associated with rigidity. A seminal study by Ackerman et al. (2010) tested whether the sensation of hardness would influence people’s perception of these attributes in social situations. They showed that, when asked to evaluate the personality traits of a depicted employee, people in contact with a hard block of wood (thus exposed to a hard sensation) judged the employee to be more rigid than those in contact with a soft block of wood (thus exposed to a soft sensation). In addition, people who made their judgment while sitting on a hard-surface rather than on a cushioned stool judged the employee to be more stable and less emotional.

Although the above findings suggest that physical interactions with the tactile sensation of hardness influences participants’ social impressions, it is less clear whether the sensation of hardness can have a similar effect on cognitive functions via metaphorical associations. Kim (2015) provided initial evidence that the sensation of hardness can shift people’s rigidness in thinking. Using standard tests of creativity, Kim found that people were more likely to be divergent in their creativity (i.e., less rigid in their thinking) when they experienced a soft sensation (i.e., when squeezing a soft ball) but convergent in their creativity (i.e., more rigid in their thinking) when they experienced a hard sensation (i.e., squeezing a hard ball). Interesting as the results are, questionnaire-based standard tests are not ideal tools to explore whether and how cognitive functions can be influenced by the tactile sensation of hardness, because participants might understand each question in a questionnaire differently and avoid reporting some points that they did not want to report. Questionnaires also lack ecological validity and were not flexible. In addition, the manipulation of squeezing a soft vs. hard ball could have invited many confounds. For instance, it is possible that the observed effect could have been caused by other sensorimotor simulation, such as the strength applied to the ball (e.g., greater strength is needed to squeeze a hard ball) or the ease of squeezing a soft ball (e.g., a soft ball is more pliable, and therefore, easier to squeeze).

To explore more thoroughly whether the tactile sensation of hardness affects cognitive functions via metaphorical association, we used the metaphorical links between hardness/softness and rigidity/flexibility in Chinese. In Chinese, “hard” is associated with rigidity. For instance, rigid truths mean hard principles in Chinese (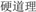). In contrast, “soft” is associated with flexibility. For instance, the proverb “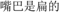, 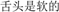
” (lit., “the mouth is flat; the tongue is soft” means that people should be flexible in their communication). In addition, softness and flexibility are usually used together in Chinese as in 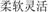 (lit., “soft and flexible”). Hence, hardness is metaphorically associated with rigidity, whereas softness is metaphorically associated with flexibility.

As they are metaphorically associated with hardness and softness, rigidity and flexibility can also describe the manner in which certain cognitive functions are performed. A well-functioning memory system, for instance, encodes and retrieves information in a rigid manner when individuals require accurate memories in order to avoid the inconvenience of forgetting or false memories (Tanila et al., 1997). Of course, rigidness may be just one of mechanisms underlying the memory system. To update existing memories and avoid the memory loss, the system would also rely on the mechanisms of plasticity and reliability. Thus, while other types of memories, e.g., episodic memories and imagining possible future events, may mainly require flexibility and creativity mechanisms (Schacter and Addis, 2009; Spreng et al., 2009), remembering words for a subsequent memory test requires the rigid mechanism.

However, creativity requires people to think in a more flexible manner in order to view a problem with a new perspective, reveal hidden patterns, or generate innovative solutions (Zabelina and Robinson, 2010).

Of course, it is possible for a cognitive function to involve both manners of thinking. Analogical reasoning, for instance, requires people to have a rigid understanding of a problem (i.e., the source) and then to map that problem to a target using a flexible manner of thinking, so that is easier to understand (Sternberg, 1977; Sowa and Majumdar, 2003; Viskontas et al., 2004). The analogy between the structure of an atom and the solar system, for example, initially requires rigid thinking about the relationship between the nucleus and the electrons and then a more flexible search for a target (the solar system). Thus, if hardness experience affects cognitive function via a metaphorical association, we should expect hard and soft bodily stimulation to affect memory, creativity, and analogical reasoning in different ways. In particular, we would expect hard (rather than soft) bodily stimulation to promote explicit memory and soft (rather than hard) bodily stimulation to facilitate creativity. However, we would expect hardness stimulation to have little or no effect on analogical reasoning, as the rigidity and flexibility effects would cancel each other out, a hypothesis we tested in this study’s two experiments.

In the experiments, we manipulated hardness bodily stimulation by having participants sit on either a hard-surface (hard condition) or a cushioned stool (soft condition) while they performed different cognitive tasks. We used three tasks that engage different cognitive functions: a memory-recall task (requiring a rigid thinking style for successful performance), a Chinese-riddle task (requiring a creative and flexible thinking style), and an analogical-reasoning task (requiring both rigid and creative thinking styles). In Experiment 1, participants were asked to perform a memory-recall task in which they memorized and recalled a list of words (León-Carrión et al., 2010). As the task mainly involved the cognitive function of memorization, we hypothesized hard bodily stimulation would be more likely to enhance task performance than soft bodily stimulation. Experiment 2 employed two tasks: a Chinese-riddle task, in which creative and flexible thinking was required for solutions, and an analogical-reasoning task that required participants to think in both a rigid and flexible manner (Kumar and Kumari, 1988; Beversdorf et al., 1999). We hypothesized that participants would perform better at solving the Chinese riddles if they sat on a cushioned rather than a hard-surface stool but would perform the analogical reasoning task at the same level, regardless of the stimulation condition.

## Experiment 1

### Method

#### Participants

Forty-five Mandarin-speaking students (29 women; mean age = 20.0, *SD* = 2.2) were recruited from South China Normal University, Guangzhou, China to participate in the experiment. None of them reported having a language disorder and all had normal or corrected-to-normal vision. Each participant was offered a small monetary reimbursement for participating in the study. All participants gave written informed consent in accordance with the Declaration of Helsinki. The study was approved by the Ethics Review Board of School of Psychology, South China Normal University.

#### Materials

We used two groups of stools to manipulate participants’ sensations of hardness, which were identical except that one group had hard surfaces and the other had cushioned ones (see **Supplementary Figure S1** for a sample of hard-surface stool).

The memory task consisted of 36 two-character Chinese words (see **Supplementary Table 1**). A pretest was administered to 25 participants who did not take part in the main experiment. Their ratings of the familiarity of the words on a 5-point scale ranging from 1 (*extremely unfamiliar*) to 5 (*extremely familiar*) showed that all of the words had a familiarity score greater than 4.5.

It is possible that these words may have a general tendency to be associated with the hard or soft sensation, and the hard-surface or cushioned stool may thus prime the recall of these words. To rule out this possible priming effect, we recruited another 20 participants to rate the association between each of the test words and hardness/softness on a 5-point Likert scale (1 indicated extremely soft while 5 indicated extremely hard). A one-sample *t-test* showed that the test words as a whole were not associated with either the hard or soft sensation (*M* = 3.07, *SD* = 0.56, *t*(35) = 0.76, *p* = 0.454, Cohen’s *d* = 0.13).

#### Procedure

Participants were randomly assigned to one of the two hardness conditions and tested individually in a quiet cubicle. Depending on the condition, participants were seated on either hard-surface or cushioned stools naturally using their comfortable postures. They were then given a clipboard with a sheet of paper containing 36 words. The clipboard was placed on an office table in front of the participants. The participants were allowed 2 minutes to memorize the words. At the end of the memorization phase, the paper was collected and the participants took a 10-min break. Then, they recalled the words by writing them down on a new sheet of paper. No time limit was imposed on the recall phase. During the entirety of the experiment, the participants remained seated on the same stool. None of the participants reported any awareness of the hardness manipulation during the debriefing at the end of the experiment. In addition, previous studies have reported that body postures affected participants’ cognitive performances (Stepper and Strack, 1993). However, we did not observe any systematic differences of body postures between hard and soft groups in this experiment.

### Results and Discussion

**FIGURE 1:**
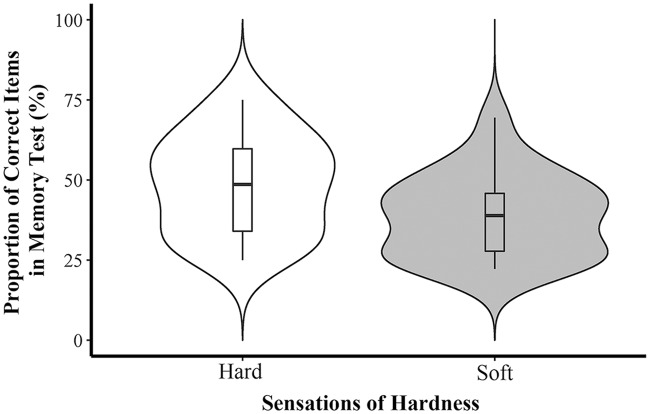
**Proportion of correct answers in recall task in Experiment 1.** The white and gray areas display the data’s probability density; the thick horizontal lines inlayed into the box correspond to medians; the lower and upper “hinges” of the box represent the first and third quartiles; and whiskers extends from the corresponding hinge to the highest/lowest value that is within 1.5× interquartile range of the hinge (subsequent figures also follow this layout).

We calculated the proportion of correctly recalled words out of the 36 test words. Given that none of the participants’ scores fell beyond ±3 *SDs*, all participants’ data were included in the analysis. An independent-samples *t*-test showed that participants sitting on the hard-surface stool correctly recalled more words (*M* = 0.49, *SD* = 0.15) than those who sat on the cushioned stool (*M* = 0.39, *SD* = 0.13) (*t*(43) = 2.35, *p* = 0.023, Cohen’s *d* = 0.70) (see **Figure 1**). These results suggest that sitting on a hard-surface stool, as compared to a soft one, facilitated participants’ memorization. This finding suggests that hardness stimulation affects cognitive functions that underlie memory recall. In Experiment 2, we tested whether hardness stimulation affects creativity and analogical reasoning.

## Experiment 2

### Method

#### Participants

Another sample of 45 participants (32 women; mean age = 20.1, *SD* = 2.4) from the same population as those in Experiment 1 were paid to take part in the experiment. The study was approved by the Ethics Review Board of School of Psychology, South China Normal University.

#### Materials

The same stools from Experiment 1 were used. To construct the Chinese riddle test, we selected 10 riddles via a Chinese search engine (wenku.baidu.com). Each riddle contained orthographic and/or semantic cues to the answer (a Chinese character). For example, question: 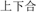 (connect the upper [i.e., 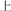] and lower [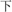]); answer: 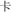 (card) (see **Supplementary Table 2**). A pilot test with 8 participants (who did not take part in the main experiment) showed that on average, they correctly solved 5.9 out of the 10 riddles. The analogical reasoning test consisted of 28 questions. The stem of each question consisted of a Chinese word pair which exhibited a logical relationship (e.g., 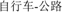 [bicycle-road]); the options were four word pairs, one of which expressed the same logical relationship as the one provided in the stem (e.g., 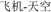 [aircraft-sky]) (see **Supplementary Table 3**).

#### Procedure

As in Experiment 1, participants were randomly assigned to one of the two hardness conditions and were seated on the corresponding stool (hard-surface or cushioned) throughout the experiment. The two tests (Chinese riddles and analogical reasoning) were carried out sequentially, with their order counterbalanced across participants. For the Chinese riddle test, participants were given a paper-and-pencil questionnaire consisting of ten riddles, and allowed 5 min to solve as many riddles as possible. For the analogical reasoning test, participants were given a paper-and-pencil questionnaire consisting of 28 analogical reasoning questions and instructed to answer as many questions as possible within 5 min. Participants were asked to remain seated until they had finished both tests. None of the participants reported an awareness of the hardness manipulation and showed any special body postures.

### Results and Discussion

The proportion of correctly answered items out of all test items (i.e., 10 items in riddle test and 28 items in analogical reasoning test) on each test was analyzed by independent *t-*tests. One participant was excluded from the analysis of the Chinese riddle scores because his/her score exceeded 3 *SD*s. All participants were included in the analysis of the analogical reasoning scores^1^.

**FIGURE 2:**
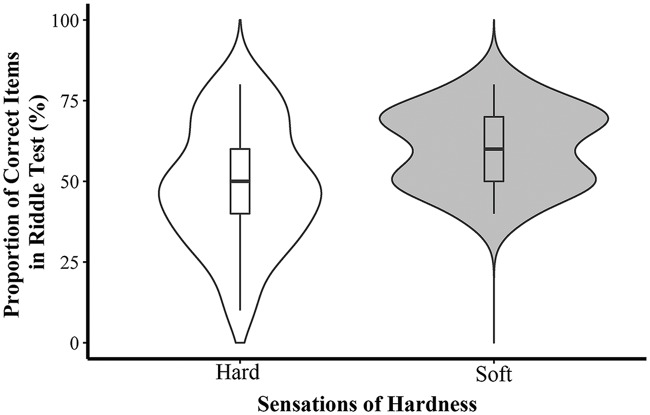
**Proportion of correct answers in Chinese riddle task in Experiment 2**.

**FIGURE 3:**
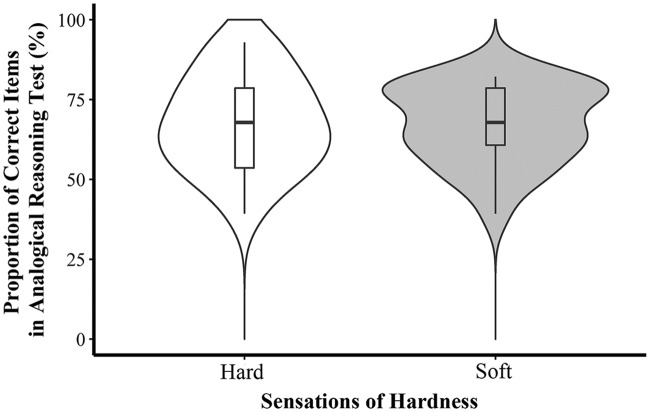
**Proportion of correct answers in analogical reasoning task in Experiment 2**.

The results of the Chinese riddle test showed that participants’ performance was better when they were seated on a soft stool (*M* = 60.45, *SD* = 12.14) than when they were seated on a hard-surface stool (*M* = 48.57, *SD* = 19.05) (*t*(41) = 2.45, *p* = 0.019, Cohen’s *d* = 0.74). The *t*-test of the scores on the analogical reasoning test showed that hardness did not affect participants’ performance (*M*_soft_ = 67.24, *SD* = 12.26; *M*_hard_ = 67.69, *SD* = 15.05; *t*(42) = -0.11, *p* = 0.913, Cohen’s *d* = -0.03) (see **Figures 2** and **3**).

These results confirmed our hypothesis that soft bodily stimulation promotes creativity/flexibility during the Chinese riddle task via a metaphorical association between softness and flexibility. In the analogical reasoning task, soft stimulation might have improved participants’ performance by promoting flexible thinking in the search for an analogical target; similarly, the hard stimulation might have improved performance through rigid thinking in relation to the source problem, thus cancelling out any possible effect by the soft simulation.

## General Discussion

In the current study, we tested whether the tactile sensation of hardness would affect participants’ cognitive functions (i.e., memory, creativity, and analogical reasoning) via metaphorical associations. The results of Experiment 1 showed that hard bodily stimulation enhanced participants’ memory: the participants who sat on a hard-surface stool accurately recalled more words in a memory test than those who sat on a cushioned stool. Experiment 2 revealed that soft bodily stimulation promoted participants’ creativity: participants who sat on a soft stool correctly solved more Chinese riddle questions than those who sat on a hard stool. Hardness/softness stimulation, however, does not bias analogical reasoning, which presumably requires both rigid and flexible thinking. This, together with previous findings, confirms the embodied cognition proposition that the interplay between body and environment can affect how the mind works (Lakoff and Johnson, 1980b; Barsalou, 2008; Landau et al., 2010).

Many studies have reported that performance on memory tests is enhanced when the testing is conducted in the same setting as the learning (Godden and Baddeley, 1975; Smith et al., 1978; Smith, 1979). The current study extends this finding by suggesting that memory is improved when the learning and testing settings lead to a bodily state that is metaphorically congruent with the memory task (e.g., a hard stimulation leading to rigid thinking in the performance of a memory task). Similarly, our research also suggests that an environment that creates “soft” feelings, which is metaphorically congruent with the Chinese riddle task, can lead to better performance on creativity tasks (Kim, 2015). Thus, these results support our hypotheses that hardness would affect participants’ cognitive functions via metaphorical associations.

The effects of hardness/softness with respect to cognitive functions occur via metaphorical association. As mentioned previously, people associate hardness with rigidness and softness with flexibility. For example, we use the term “hard science” to refer to the natural sciences, which require rigid adherence to a set of rules and methods in their quantitative research studies, and “soft science” to refer to the social sciences and humanities, which tend to rely more on argumentation and qualitative research methods. These metaphorical uses of *hard* and *soft* imply metaphorical links between the tactile experience of hardness and softness and the extent of rigidity in thinking. In the current study, hard feelings primed rigidity and its activation further facilitated the participants’ performance of a memory task than did soft feelings, because memory is metaphorically associated with rigidity. Similarity, soft feelings primed flexibility that facilitated the participants’ performance on the Chinese riddle task.

The current findings further extend the role of metaphor in creativity. Philosophers note that metaphors can create novel features of an object or situation, known as the creativity of metaphor. Take the metaphor, “There are some days the happy ocean lies// Like an unfingered harp, below the land.” for instance. This metaphor emphasizes the sun’s reflection on the ripples of a peaceful ocean and provides us a new view of the ocean (Indurkhya, 1999). The results of the Experiment 2 extend the premise that hardness affects creativity in cognitive function, indicating that metaphors not only create novel features directly, but may also create special metaphorical associations to improve people’s creativity.

Thus, these findings suggest that bodily stimulation plays an important role in cognitive function, specifically, the manner in which people acquire new information or search for and retrieve existent information for problem solving (Messick, 1976; See Sternberg and Grigorenko, 1997; Kozhevnikov, 2007 for reviews). Prior research has suggested that cognitive function may be affected by external sensory experience. Mehta and Zhu (2009) presented evidence that a red and a blue visual background exerted different influences on the way people performed cognitive tasks. A red background biased people’s attention towards details (e.g., leading to better word recall), whereas a blue background enhanced people’s creativity (e.g., leading to more creative use of an object). These results are parallel with our findings that hard bodily stimulation enhanced memory performance, whereas soft bodily stimulation promoted creativity. Mehta and Zhu (2009) argued that these color effects on cognitive functions are caused by associations: the red color is associated with danger and mistakes, and therefore raises people’s vigilance, which in turn, leads to better comprehension of details; the blue color, in contrast, is associated with openness, peace, and tranquility, which in turn, facilitates creativity in problem solving.

Although these associations may be common, it is unclear how they explain our results. Our daily experience offers a multitude of direct (i.e., sensorimotor perception-cognitive function) and indirect (sensorimotor perception-metaphorical association-cognitive function) associations between sensorimotor perceptions and conceptual meanings. For example, the association between warmth and prosocial feelings is mainly direct. Warmth is necessary for survival and it can make people feel safe. Hence, people generally associate warmth with prosocial feelings. When participants encountered social exclusion in one study, they preferred warm food and drink to reduce their bad feelings, in comparison to those who did not experience such exclusion (Zhong and Leonardelli, 2008).

Nevertheless, the effects of hardness on cognitive functions are likely to be based on indirect associations. In our daily lives, hardness stimulation does not have any direct functions or obvious meanings in memorization or creativity. Rarely do people explicitly experience better memory or a spur of creativity by having a particular tactile experience. Then, what are mechanisms underlying the observed associations? One interesting possibility is linguistic/metaphorical association, i.e., indirect association (Lakoff and Johnson, 1980a,b; Gibbs, 1994). Metaphor is the fundament of the conceptual system that is used to understand abstract concepts through superficially dissimilar concrete concepts. The concrete concepts, e.g., hardness and softness, come from individuals’ interactions with the environment. Conceptual metaphors help individuals adopt these concrete concepts as analogical framework for supporting their understanding of abstract concepts, e.g., memory and creativity (Landau et al., 2011). For instance, as the word “hard” implies rigidity (in Chinese at least), sitting on a hard-surface stool may instill some feeling of rigidness, which may provide metaphorical support for the memory system, and prime the formation of more rigid memories that are less susceptible to interference or forgetting. Similarly, as the word “soft” implies flexibility, a cushioned stool might leave the impression of physical flexibility, which metaphorically facilitates flexibility in thinking, thereby leading to increased creativity in the Chinese riddle task. Such a linguistically mediated metaphorical association is in line with recent research and highlights the importance of linguistic associations in cognitive processing (e.g., Andrews et al., 2009; Connell and Lynott, 2013).

Another potential account in line with indirect metaphorical account is social embodiment, which holds that individuals’ performance is modulated by the compatibility of bodily states and cognitive states (Barsalou et al., 2003; Niedenthal, 2007; Gallese and Sinigaglia, 2011). In the current experiments, when participants sat on a hard-surface stool to learn a list of words, their tactile sensations (i.e., hardness) were compatible with their cognitive states (i.e., encoding words rigidly). Then, participants sitting on a hard-surface stool had better performance in the recalling test than those sitting on a soft stool. Similarly, when participants sat on a soft stool to solve the Chinese riddle question, their tactile sensation (i.e., softness) was compatible with their cognitive states (i.e., adopting flexible and creative thinking). As a result, the soft stool facilitated participants’ performance in the Chinese riddle test than the hard one. Given that the current study did not intend to distinguish the underlying mechanisms of the observed effects but mainly focused on verifying the existence of such effects, future studies may distinguish the metaphorical and embodied accounts of the observed effects.

The results of the current experiments also verify the relationship between embodiment and metaphor. Barsalou (1999) proposed that perceptual symbol systems represent knowledge for cognitive processing. Gibbs and Berg (1999) further distinguished embodiment (e.g., bodily experience) and metaphor in these symbols of knowledge representation. They hold that knowledge is represented as perceptual symbols that are inherently structured by metaphor for the most part. Hence, metaphor may organize perceptual symbol systems. In the current study, memory and creativity were also grounded in perceptual experience and bodily states, such as tactile sensation. Metaphors, e.g., hardness as rigidity whereas softness as flexibility, help to ground less concrete concepts such as rigidity and flexibility in tactile sensation. When perceptual symbols are activated (e.g., by sitting on a cushioned/non-cushioned stool), related metaphors are activated, which in turn lead to the associated abstract concepts.

Several embodied processes may interact with a cognitive function, such as analogical reasoning in the current study. Analogical reasoning requires people to understand a problem using rigid thinking, and then to use flexible thinking to transfer information of the problem to a target (Sternberg, 1977; Sowa and Majumdar, 2003; Viskontas et al., 2004). Hence, hard and soft stools affected the participants’ performance in different ways, resulting in the absence of a metaphorical effect in the analogical reasoning test. However, this interpretation is merely our hypothesis. Follow-up studies should adopt different types of analogical reasoning tasks that emphasize hard and soft metaphorical associations differently to test our interpretation. In addition, further studies should examine whether such multiple embodied processes also interact with other cognitive functions, such as decision-making and moral judgment.

Whether comfort of stools and participants’ mood modulate the observed findings is not clear. First, soft seats usually produce more even pressure distributions than hard ones, which makes soft seats more comfortable than hard ones. However, this effect is also modulated by individuals’ adipose tissue. Lean individuals would also produce high-pressure peaks on very soft seats (Kolich, 2008). However, as we did not measure the participants’ rating on the comfort of hard and soft stools, we do not know whether the observed effects are modulated or even induced by the comfort of stools. A second potential mechanism underlying the observed effect may be the participants’ mood. Mood affects individuals’ memory, such that a positive mood facilitated participants’ learning for new brand names than a neural one (Lee and Sternthal, 1999). Unfortunately, we did not measure the participants’ mood when they sat on hard or soft stools and thus the potential influence of mood for the observed effect is not clear. Nevertheless, these two factors are not confounding variables and may be, actually, mechanisms underlying the observed effects in this study. The tactile sensation of hardness may modulate participants’ comfort levels and/or their mood, which then in turn induces the observed effects. Given that we have found the hardness of stools modulated the participants’ memory and creativity, comfort and mood may be mediators between hardness and cognitive functions. Follow-up studies should use experimental manipulation or structural equation modeling to test these hypotheses.

Future research should also investigate whether the mechanism of metaphorical association works bi-directionally. Several studies have failed to observe a metaphorical effect of an abstract domain on a concrete domain (e.g., importance of physical weight on psychological significance) (Zhang and Li, 2012). Lee and Schwarz (2012) found that social suspicion and the perception of a fishy smell have a reciprocal effect on each other. As exposure to a fishy smell increases suspicion and undermines cooperation among people, an increase in social suspicion can similarly heighten people’s sensitivity to a fishy smell. Given this observation, it would be interesting to see whether performance in memory and creativity tasks have a reciprocal effect on the perception of hardness.

In sum, the current study found that cognitive functions, such as memory and creativity might be modulated by the bodily experience of hardness, thereby lending support to the embodiment claim that cognition employs bodily states. These findings support the embodied account and have implications for the debates between embodiment and disembodiment (Clark, 1999; Tversky and Hard, 2009; Maglio and Trope, 2012; Pavlenko, 2012; Pulvermüller, 2013; Pulvermüller and Garagnani, 2014).

## Author Contributions

RW, ZL, and JX designed the experiment and analyzed the data. ZL collected the data and prepared the dataset for analyses. JX analyzed the data and wrote the first draft of the manuscript. ZGC, RW, and JX interpreted the data and revised the draft critically.

## Conflict of Interest Statement

The authors declare that the research was conducted in the absence of any commercial or financial relationships that could be construed as a potential conflict of interest.

## Footnotes

1 In another analysis of the analogical reasoning test, we also removed the participant who had been excluded from the riddle test. The result of the analogical reasoning test remained the same as reported above.
