# Supplementary material for: Remember Hard But Think Softly: Metaphorical Effects of Hardness/Softness on Cognitive Functions
Source: Front Psychol. 2016 Sep 12;7:1343. doi: 10.3389/fpsyg.2016.01343 (PMC5018472; doi:10.3389/fpsyg.2016.01343)
Supplement: Supplementary file 1 [file Table_1.DOCX]

Supplementary Material

Remember Hard but Think Softly: Metaphorical Effects of Hardness/Softness on Cognitive Functions

**Jiushu Xie^1, 2^, Zhi Lu^1, 2^, Ruiming Wang*^1, 2^, Zhenguang G. Cai^3, 4^**

^1^ Center for Studies of Psychological Application, School of Psychology, South China Normal University, Guangzhou, China

^2^ Guangdong Provincial Key Laboratory of Mental Health and Cognitive Science, South China Normal University, Guangzhou, China

^3^ School of Psychology, University of East Anglia, Norwich, United Kingdom

^4^ Department of Experimental Psychology, University College London, London, United Kingdom

*** Correspondence**: Ruiming Wang, Center for Studies of Psychological Application, School of Psychology, South China Normal University, Guangzhou, 510631, Guangdong Province, China.

E-mail: wangrm@scnu.edu.cn.

# Supplementary Tables

Table 1. Chinese words used in the memory recall task of Experiment 1

| Chinese Words | English Translation | Chinese Words | English Translation | Chinese Words | English Translation |
| --- | --- | --- | --- | --- | --- |
| 明亮 | Brightness | 寒冷 | Cool | 光亮 | Light |
| 喧闹 | Noise | 酸痛 | Ache | 橙色 | Orange |
| 寺庙 | Temple | 疼痛 | Pain | 麻辣 | Spicy |
| 凉快 | Cold | 迷彩 | Camouflage | 别墅 | Villa |
| 细胞 | Cell | 柔软 | Soft | 油腻 | Fatness |
| 冰冷 | Iciness | 耀眼 | Dazzle | 难吃 | Unsavory |
| 透明 | Transparent | 美味 | Delicious | 悦耳 | Musical |
| 温暖 | Warm | 集团 | Group | 语法 | Grammar |
| 杂志 | Magazine | 吼叫 | Howl | 黑暗 | Black |
| 安静 | Quiet | 寂静 | Silent | 打鼾 | Snore |
| 潮湿 | Humid | 鲜艳 | Colorful | 悠扬 | Melodious |
| 模糊 | Blur | 邮件 | Mail | 坚硬 | Rigidity |

Table 2. Chinese riddles used in the Chinese riddle task of Experiment 2^[[1]](#footnote-1)^

| Number | Chinese riddles with answers in parentheses  (The English translations follow the riddles and answers.) |
| --- | --- |
| 1 | 凶横 (答案: 区) |
|  | Horizontal atrocity (凶). (Answer: area) |
| 2 | 九点 (答案: 丸) |
|  | Nine (九) points. (Answer: pellet) |
| 3 | 镜中人 (答案: 入) |
|  | Man (人) in the mirror. (Answer: enter) |
| 4 | 上下合 (答案: 卡) |
|  | Connect the upper (上) and lower(下). (Answer: card) |
| 5 | 一箭穿心 (答案: 必) |
|  | An arrow through the heart (心). (Answer: certain) |
| 6 | 打断念头 (答案: 心) |
|  | Give up an idea (i.e., remove念’s head). (Answer: heart) |
| 7 | 休要丢人现眼 (答案: 相) |
|  | Don’t make a fool of yourself (i.e., replace the left part of 休with 目). (Answer: phase) |
| 8 | 大雨落在横山上 (答案: 雪) |
|  | Heavy rain (雨) fell on the horizontal mountain(山). (Answer: snow) |
| 9 | 一字生的巧, 四面八只脚 (答案: 井) |
|  | One well-born character, eight feet on four sides (Answer: well) |
| 10 | 一点一横长, 一撇到南洋, 南洋有个人, 只有一寸长 (答案: 府) |
|  | A dot and a long stroke, a left-falling stroke to Southeast Asia. There was a man (人), only one inch (寸) high, in Southeast Asia (Answer: mansion) |

Table 3. Questions used in the analogical reasoning task of Experiment 2

| Number | Analogical reasoning questions with answers in parentheses  (English translations are below the questions and answers) |
| --- | --- |
| 1 | 问题：义工: 职员 |
|  | A. 球迷: 球员 B. 学生: 教师 C. 初学者: 生手 D. 志愿者: 雇员 |
|  | 答案: D |
|  | Question: Volunteer vs. staff |
|  | A. soccer fans vs. footballer B. student vs. teacher C. beginner vs. layman D. volunteer vs. employee |
|  | Answer: D |
| 2 | 问题：努力: 成功 |
|  | A.生根: 发芽 B.耕耘: 收获 C.城市: 乡村 D.原告: 被告 |
|  | 答案: B |
|  | Question: Endeavor vs. success |
|  | A. to root vs. to sprout B. cultivation vs. harvest C. city vs. countryside D. the prosecutor vs. the defense |
|  | Answer: B |
| 3 | 问题：锯子: 木头 |
|  | A. 窗户: 玻璃 B. 刀片: 铅笔 C.剪刀: 布匹 D.牙膏: 牙刷 |
|  | 答案: C |
|  | Question: Saw vs. wood |
|  | A. window vs. glass B. blade vs. pencil C. scissors vs. cloth D. toothpaste vs. toothbrush |
|  | Answer: C |
| 4 | 问题：射击: 手枪 |
|  | A. 投掷: 石头 B. 个性: 温和 C. 小桥: 流水 D. 追求: 光明 |
|  | 答案: A |
|  | Question: Shoot vs. gun |
|  | A. throw vs. stone B. personality vs. gentle C. bridge vs. water D. pursuit vs. light |
|  | Answer: A |
| 5 | 问题：自行车: 公路 |
|  | A. 河流: 芦苇 B. 飞机: 天空 C. 城市: 农村 D. 家具: 灯具 |
|  | 答案: B |
|  | Question: Bicycle vs. street |
|  | A. river vs. reed B. airplane vs. sky C. city vs. countryside D. furniture vs. lamps |
|  | Answer: B |
| 6 | 问题：面粉: 小麦 |
|  | A. 大米: 稻谷 B. 桔子: 葡萄 C. 饼干: 面粉 D. 罐头: 菠萝 |
|  | 答案: A |
|  | Question: Flour vs. wheat |
|  | A. rice vs. grain B. orange vs. grapes C. biscuit vs. flour D. cans vs. pineapple |
|  | Answer: A |
| 7 | 问题：馒头: 食物 |
|  | A. 食品: 饼干 B. 头: 身体 C. 手: 食指 D. 钢铁: 金属 |
|  | 答案: D |
|  | Question: Bun vs. food |
|  | A. food vs. biscuit B. head vs. body C. hand vs. index finger D. iron vs. metal |
|  | Answer: D |
| 8 | 问题：水: 龙头 |
|  | A. 电: 电线 B. 电: 开关 C. 电: 发电 D.电: 电灯 |
|  | 答案: B |
|  | Question: Water vs. faucet |
|  | A. electricity vs. electric wire B. electricity vs. switch C. electricity vs. power generator D. electricity vs. lamps |
|  | Answer: B |
| 9 | 问题：稠密: 稀疏 |
|  | A. 宽敞: 明亮 B. 伟大: 平凡 C. 词语: 词汇 D.酷热: 炎热 |
|  | 答案: B |
|  | Question: Thick vs. thin |
|  | A. spacious vs. bright B. great vs. ordinary C. words vs. vocabulary D. hot vs. broiling |
|  | Answer: B |
| 10 | 问题：茄子: 蔬菜 |
|  | A. 马铃薯: 土豆 B. 工人: 农民 C. 猫: 动物 D. 花菜: 大白菜 |
|  | 答案: C |
|  | Question: Eggplant vs. vegetable |
|  | A. potato vs. murphy B. worker vs. farmer C. cat vs. animal D. cauliflower vs. Chinese cabbage |
|  | Answer: C |
| 11 | 问题：马: 牲畜 |
|  | A. 蜘蛛: 琥珀 B. 南瓜: 瓜农 C. 铁: 金属 D.布: 纺织 |
|  | 答案: C |
|  | Question: Horse vs. livestock |
|  | A. spider vs. amber B. pumpkin vs. farmer C. iron vs. metal D. cloth vs. weave |
|  | Answer: C |
| 12 | 问题：皮带: 带扣 |
|  | A. 鞋子: 鞋带 B. 子弹: 步枪 C. 手套: 围巾 D. 帽子: 头发 |
|  | 答案: A |
|  | Question: Belt vs. buckle |
|  | A. shoes vs. shoelace B. bullet vs. rifle C. gloves vs. scarf D. hat vs. head |
|  | Answer: A |
| 13 | 问题：啤酒: 杯子 |
|  | A. 漏斗: 木桶 B. 象棋: 棋盘 C. 电灯: 插座 D. 眼镜: 镜盒 |
|  | 答案: D |
|  | Question: Beer vs. glass |
|  | A. funnel vs. barrel B. chess vs. chessboard C. lamp vs. socket D. glasses vs. eyeglass case |
|  | Answer: D |
| 14 | 问题：抱怨: 埋怨 |
|  | A. 胆怯: 怯弱 B. 真挚: 虚假 C. 精确: 近似 D. 隐蔽: 公开 |
|  | 答案: A |
|  | Question: Complain vs. blame |
|  | A. timid vs. coward B. sincere vs. hypocritical C. accurate vs. approximate D. implicit vs. explicit |
|  | Answer: A |
| 15 | 问题：香蕉: 水果 |
|  | A. 高山: 天山 B. 树枝: 树木 C. 黄梨: 香梨 D. 桌子: 家具 |
|  | 答案: D |
|  | Question: Banana vs. fruit |
|  | A. mountain vs. Mt. Tianshan B. branch vs. tree C. yellow pear vs. fragrant pear D. table vs. furniture |
|  | Answer: D |
| 16 | 问题：香瓜: 木瓜 |
|  | A. 绿豆: 豌豆 B. 松鼠: 树林 C. 鲨鱼: 鲸鱼 D. 家具: 灯具 |
|  | 答案: A |
|  | Question: Muskmelon vs. papaya |
|  | A. mung beans vs. peas B. squirrel vs. woods C. shark vs. whale D. furniture vs. lamp |
|  | Answer: A |
| 17 | 问题：赞美: 漂亮 |
|  | A. 你好: 回应 B. 责问: 悔改 C. 安慰: 工作 D. 祝愿: 平安 |
|  | 答案: D |
|  | Question: Praise vs. beautiful |
|  | A. hello vs. response B. question vs. repent C. comfort vs. work D. wish vs. safe |
|  | Answer: D |
| 18 | 问题：(____) : 没有人所有的: (____) |
|  | A. 有些更多 B. 每个人没有 C. 很多从不 D. 一些总是 |
|  | 答案: B |
|  | Question: (____) vs. nobody, all vs. (____) |
|  | A. several vs. more B. everybody vs. none C. many vs. never D. some vs. always |
|  | Answer: B |
| 19 | 问题：羊毛: 珍珠羊: ( ) |
|  | A. 奶酪 B. 牛奶 C. 山羊 D. 牡蛎 |
|  | 答案: D |
|  | Question: Wool vs. pearl vs. sheep vs. ( ) |
|  | A. cheese B. milk C. goat D. oyster |
|  | Answer: D |
| 20 | 问题：(____) : 生病汗水: (____) |
|  | A. 医生 成功 B. 药片 夸奖 C. 发高烧 努力 D. 温度计 上司 |
|  | 答案: C |
|  | Question: (____) vs. illness, sweat vs. (____) |
|  | A. doctor success B. pill praise C. fever endeavor D. thermometer superior |
|  | Answer: C |
| 21 | 问题：大雁: 南飞 |
|  | A. 企鹅: 迁徒 B. 苍鹰: 高飞 C. 乌龟: 冬眠 D. 蚂蚁: 搬家 |
|  | 答案: C |
|  | Question: Wild goose vs. fly south |
|  | A. penguin vs. migrate B. eagle vs. soar high C. tortoise vs. hibernate D. ants vs. move |
|  | Answer: C |
| 22 | 问题：老师: 学生 |
|  | A. 教师: 职工 B. 编辑: 读者 C. 师傅: 学徒 D. 演员: 经纪人 |
|  | 答案: C |
|  | Question: Teacher vs. students |
|  | A. teacher vs. staff B. editor vs. reader C. master vs. apprentice D. actor vs. agent |
|  | Answer: C |
| 23 | 问题：森林: 树木 |
|  | A. 山脉: 山 B. 花: 菊花 C. 头: 身体 D. 身体: 身躯 |
|  | 答案: A |
|  | Question: Forest vs. tree |
|  | A. mountain range vs. mountain B. flower vs. chrysanthemum C. head vs. body D. body vs. bulk |
|  | Answer: A |
| 24 | 问题：物质: 意识 |
|  | A. 发展: 运动 B. 权利: 义务 C. 成功: 勤学 D. 劳动: 懒惰 |
|  | 答案: B |
|  | Question: Substance vs. consciousness |
|  | A. development vs. movement B. rights vs. duty C. success vs. diligence D. labor vs. indolence |
|  | Answer: B |
| 25 | 问题：皮革: 铁 |
|  | A. 有弹性的: 重的 B. 结实的: 固定的 C. 弯曲的: 平滑的 D. 坚韧的: 坚硬的 |
|  | 答案: D |
|  | Question: Leather vs. iron |
|  | A. inflexible vs. heavy B. strong vs. firm C. curly vs. smooth D. tough vs. hard |
|  | Answer: D |
| 26 | 问题：白天: 黑夜 |
|  | A. 冬天: 春天 B. 上山: 下山 C. 东南: 西北 D. 月圆: 月亏 |
|  | 答案: D |
|  | Question: Day vs. night |
|  | A. winter vs. spring B. uphill vs. downhill C. southeast vs. northwest D. the full moon vs. the waning moon |
|  | Answer: D |
| 27 | 问题：运河: 河 |
|  | A. 灌木: 草地 B. 公园: 风景 C. 大米: 寿司 D. 语言: 文笔 |
|  | 答案: B |
|  | Question: Canal vs. river |
|  | A. bush vs. lawn B. park vs. scenery C. rice vs. sushi D. language vs. style of writing |
|  | Answer: B |
| 28 | 问题：招供: 审问 |
|  | A. 诊断: 检查 B. 旅游: 问询 C. 检查: 治疗 D. 查看: 了解 |
|  | 答案: A |
|  | Question: Confession vs. interrogation |
|  | A. diagnosis vs. inspection B. travel vs. inquire C. inspection vs. treatment D. examine vs. know |
|  | Answer: A |

1. These Chinese riddle questions mainly test the structure of Chinese characters and are culture-bound. To correctly answer these questions require some knowledge of Chinese. Hence, non-Chinese speakers may be puzzled to understand some logical flows between the riddle questions and the answers. [↑](#footnote-ref-1)
