# Supplementary figures and images for: Remember Hard But Think Softly: Metaphorical Effects of Hardness/Softness on Cognitive Functions
Source: Front Psychol. 2016 Sep 12;7:1343. doi: 10.3389/fpsyg.2016.01343 (PMC5018472; doi:10.3389/fpsyg.2016.01343)

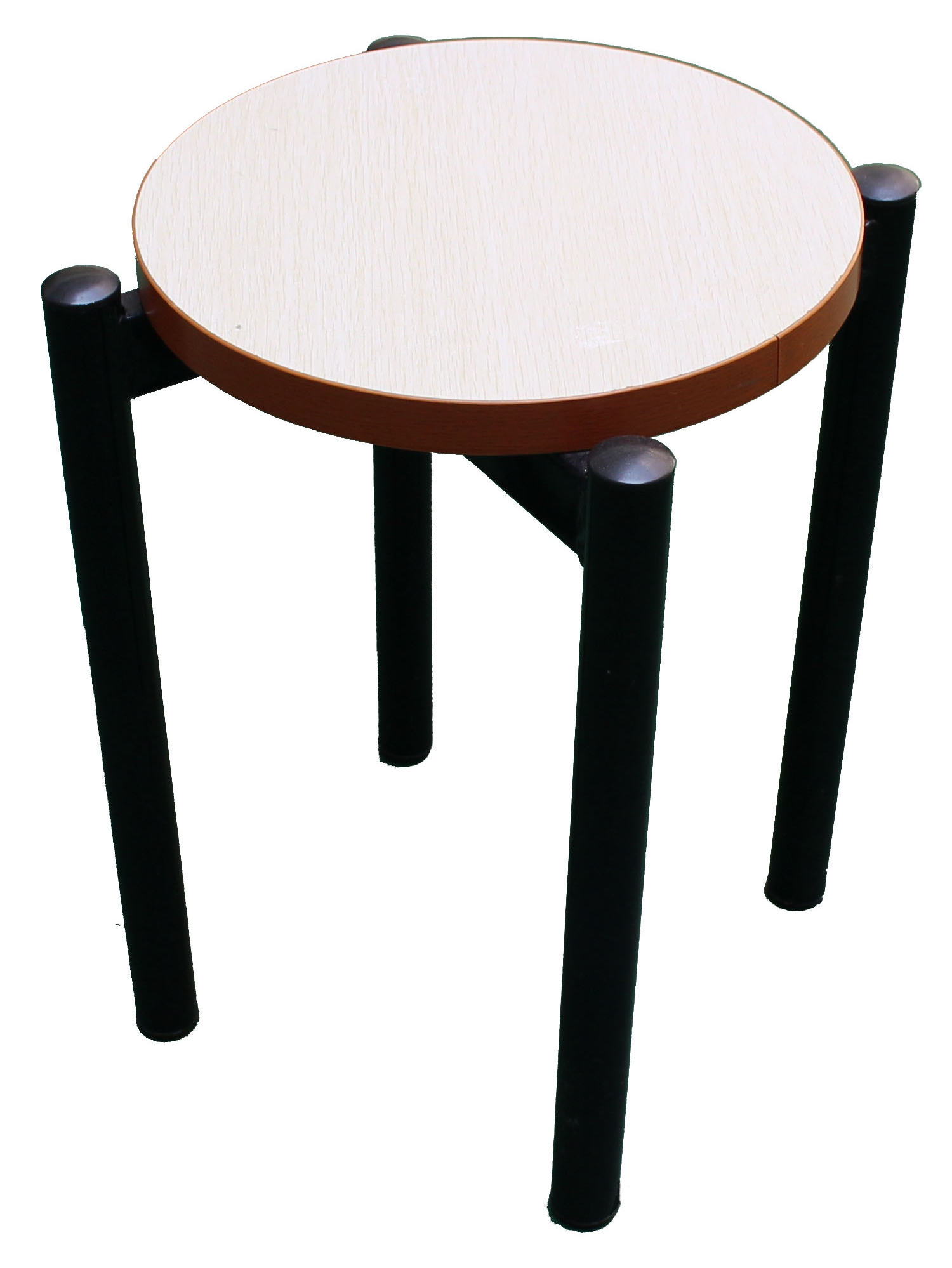

Supplement: Supplementary file 2 [file Image_1.TIF]
